# Combining PM_2.5_ Component Data from Multiple Sources: Data Consistency and Characteristics Relevant to Epidemiological Analyses of Predicted Long-Term Exposures

**DOI:** 10.1289/ehp.1307744

**Published:** 2015-02-27

**Authors:** Sun-Young Kim, Lianne Sheppard, Timothy V. Larson, Joel D. Kaufman, Sverre Vedal

**Affiliations:** 1 Department of Environmental and Occupational Health Sciences, University of Washington, Seattle, Washington, USA; 2 Institute of Health and Environment, Seoul National University, Seoul, Korea; 3 Department of Biostatistics,; 4 Department of Civil and Environmental Engineering,; 5 Department of Medicine, and; 6 Department of Epidemiology, University of Washington, Seattle, Washington, USA

## Abstract

**Background:**

Regulatory monitoring data have been the exposure data resource most commonly applied to studies of the association between long-term PM_2.5_ components and health. However, data collected for regulatory purposes may not be compatible with epidemiological studies.

**Objectives:**

We studied three important features of the PM_2.5_ component monitoring data to determine whether it would be appropriate to combine all available data from multiple sources for developing spatiotemporal prediction models in the National Particle Component and Toxicity (NPACT) study.

**Methods:**

The NPACT monitoring data were collected in an extensive monitoring campaign targeting cohort participant residences. The regulatory monitoring data were obtained from the Chemical Speciation Network (CSN) and the Interagency Monitoring of Protected Visual Environments (IMPROVE). We performed exploratory analyses to examine features that could affect our approach to combining data: comprehensiveness of spatial coverage, comparability of analysis methods, and consistency in sampling protocols. In addition, we considered the viability of developing spatiotemporal prediction models given *a*) all available data, *b*) NPACT data only, and *c*) NPACT data with temporal trends estimated from other pollutants.

**Results:**

The number of CSN/IMPROVE monitors was limited in all study areas. The different laboratory analysis methods and sampling protocols resulted in incompatible measurements between networks. Given these features we determined that it was preferable to develop our spatiotemporal models using only the NPACT data and under simplifying assumptions.

**Conclusions:**

Investigators conducting epidemiological studies of long-term PM_2.5_ components need to be mindful of the features of the monitoring data and incorporate this understanding into the design of their monitoring campaigns and the development of their exposure prediction models.

**Citation:**

Kim SY, Sheppard L, Larson TV, Kaufman JD, Vedal S. 2015. Combining PM_2.5_ component data from multiple sources: data consistency and characteristics relevant to epidemiological analyses of predicted long-term exposures. Environ Health Perspect 123:651–658; http://dx.doi.org/10.1289/ehp.1307744

## Introduction

Evidence of the association between long-term exposure to ambient PM_2.5_ (particulate matter with diameter ≤ 2.5 μm) and human health continues to accumulate (Laden et al. 2006; Miller et al. 2007; Pope et al. 2002, 2004; Puett et al. 2009) and has spurred research into understanding the role of specific PM_2.5_ chemical components (Mauderly and Chow 2008; Ostro et al. 2010; Schlesinger 2007; Vedal et al. 2013). Recent cohort studies have relied on predictions of long-term average PM_2.5_ concentrations at participant homes based on models developed from monitoring data (Eeftens et al. 2012; Paciorek et al. 2009; Sampson et al. 2011, 2013; Szpiro et al. 2010; Yanosky et al. 2009). A few additional studies have used this approach to estimate the health effects of PM_2.5_ components (Bergen et al. 2013; de Hoogh et al. 2013).

Parallel research in the statistics literature suggests that features of the monitoring data can affect the quality of the prediction models (Diggle et al. 2010; Gelfand et al. 2012) and the resulting health effect estimates (Szpiro and Paciorek 2013; Szpiro et al. 2011). Regulatory monitoring data collected and managed by government agencies are a common and useful resource for epidemiological applications. For the study of health effects of PM_2.5_ chemical components in the United States, most studies have used data from two networks: the U.S. Environmental Protection Agency (EPA) Chemical Speciation Network (CSN) and the Interagency Monitoring of Protected Visual Environment (IMPROVE) sponsored by the U.S. EPA and other agencies (Bergen et al. 2013; Ostro et al. 2010; Pope et al. 2002). However, because these monitoring networks were designed for regulatory purposes, they may not be suited to some epidemiological applications.

The University of Washington National Particle Component and Toxicity (NPACT) study was designed to investigate the associations between long-term exposure to PM_2.5_ chemical components and cardiovascular health partly based on the Multi-Ethnic Study of Atherosclerosis (MESA) cohort. NPACT collected PM_2.5_ component concentrations in the framework of an extensive cohort-focused monitoring campaign of the MESA and Air Pollution (MESA Air) study to capture fine-scale spatial variability at the residences of the MESA/MESA Air study cohort. This spatially resolved monitoring may be particularly meaningful for understanding PM_2.5_ components because many are largely affected by local sources. It will also enhance our ability to characterize within-community spatial variability in our exposure prediction models. In the original plan, the NPACT monitoring data were intended to be combined with regulatory monitoring data in exposure prediction models, similar to the approach used previously for predicting PM_2.5_ (Keller et al. 2015; Paciorek et al. 2009; Sampson et al. 2011; Yanosky et al. 2009). To meet this objective, we first needed to assess various features of the PM_2.5_ component data in order to ensure they could be combined in prediction modeling.

In this paper we compare and contrast the compatibility of the CSN and IMPROVE regulatory monitoring network data with the NPACT monitoring data within the context of the NPACT study goals. In particular, we discuss the spatial coverage of exposure monitoring, the filter analysis methods, and the sampling protocols. NPACT analyses focused on four primary pollutants: elemental and organic carbon (EC and OC), silicon, and sulfur as markers for combustion sources, crustal dust, and inorganic aerosol, respectively. Here we restrict our attention to EC and silicon, because these pollutants have been associated with adverse health outcomes (Ostro et al. 2010; Vedal et al. 2013) and they allow us to highlight similarities and differences in the features we compare.

## Methods

*Population*. The NPACT study was based on the subjects who were originally recruited in MESA and consented to MESA Air or who were directly enrolled in MESA Air. The cohort includes approximately 7,000 participants residing in six U.S. metropolitan areas: Baltimore, Maryland; Chicago, Illinois; Los Angeles, California; Minneapolis–St. Paul, Minnesota; New York City, New York; and Winston-Salem, North Carolina (Bild et al. 2002; Kaufman et al. 2012).

*Data*. NPACT monitoring data. To characterize spatial variability of exposures across participant residences, the NPACT study expanded the MESA Air exposure monitoring campaign to also measure PM_2.5_ components (Vedal et al. 2013). The MESA Air campaign focused on measuring PM_2.5_ mass and gaseous pollutant concentrations. In each city the campaign included three to seven fixed NPACT sites measuring pollutants in 2-week samples over multiple years, and approximately 50 rotating home-outdoor sites that each provided one to three 2-week samples (average of 1.8 samples) (Cohen et al. 2009). One fixed NPACT site was co-located with one CSN site in each city. Whereas the NPACT sampling for trace elements was carried out over 4 years (August 2005 through August 2009), carbon data were collected over 18 months (March 2007 through August 2008). Two-week samples for trace elements and carbon were collected on Teflon and quartz filters, respectively, in Harvard Personal Environmental Monitors (HPEMs) with a 2.5-μm cut size and pump flow rate of 1.8 L/min. PM_2.5_ components were quantified in U.S. EPA–certified labs using analysis methods consistent with those currently employed in the CSN and IMPROVE networks as described in detail by Vedal et al. (2013). In brief, trace elements were quantified using X-ray Fluorescence (XRF) (Cooper Environmental Services, Portland, OR). EC and OC were blank-corrected and quantified using the IMPROVE_A Total Optical Reflectance (TOR) method (Sunset Laboratory Inc., Tigard, OR). In addition, the NPACT study carried out comprehensive quality assurance and control procedures to minimize errors from field activities and lab analyses.

Regulatory monitoring data. The CSN and IMPROVE networks have collected 24-hr average samples of PM_2.5_ components across the United States every third or sixth day since 2000 and 1988, respectively (Hand et al. 2011; Rao et al. 2003; U.S. EPA 2004, 2005a). Monitoring sites in CSN are mostly located in urban areas to identify and control potential sources of PM_2.5_, whereas IMPROVE sites are largely deployed in rural areas to assess and regulate visibility (Hand et al. 2011; U.S. EPA 2004). From the > 300 monitoring sites in both networks combined, we selected the 99 monitoring sites within 200 km of the centers of the six MESA city regions, and downloaded from the U.S. EPA Air Quality System (AQS) database all measurements collected between 1999 and 2009. We began with 1999 because it is 1 year before the baseline screening of MESA participants. In CSN and IMPROVE, PM_2.5_ components were sampled by compliance samplers (U.S. EPA 1998). The two networks measured trace elements by XRF, including silicon and sulfur. In the CSN network, EC and OC were measured by the National Institute for Occupational Safety and Health (NIOSH) Total Optical Transmittance (TOT) or IMPROVE_A TOR method (without blank correction for both methods). In contrast, IMPROVE has only used IMPROVE_A TOR with blank correction.

Data processing. We focused on silicon and EC in this paper. We selected EC over OC because most previous epidemiological or toxicological studies that considered carbon measurements focused on EC. We selected silicon over sulfur so we could highlight interesting features of the silicon data found in our exploratory analyses. [See Vedal et al. (2013) for the full data description and exploratory analyses.] To align with NPACT’s 2-week average integrated samples, we computed averages of daily CSN/IMPROVE data for the corresponding 2-week periods centered on every other Wednesday. We log-transformed (natural log) the 2-week averages after adding 1 to approximate a normal distribution. In sensitivity analyses we found our results were insensitive to the addition of a different constant, namely 0.1 times the average of each component (data not shown).

*Features affecting between-network comparability*. We focused on spatial coverage, filter analysis protocol, and sampling protocol as factors that may influence data comparability among the CSN, IMPROVE, and NPACT networks.

Spatial coverage. Monitoring sites in the CSN and IMPROVE networks are located far from each other and typically comprise only one or a few sites in a city, whereas the NPACT monitoring sites were densely located within each MESA city region. The sparse spatial coverage of the regulatory monitoring data limits our ability to model PM_2.5_ component concentrations over space (Lippmann 2009).

Filter analysis protocol. Analytical methods for EC and OC differed within and between networks. In particular, CSN has historically used the NIOSH TOT method, whereas IMPROVE uses the IMPROVE_A TOR method. The two methods use different time/temperature analytical protocols to measure fractions of EC and OC on quartz filters. Data discrepancies resulting from these method differences have been documented (Chow et al. 2001; Malm et al. 2011). Consequently, the U.S. EPA decided to change the laboratory method for CSN sites to the IMPROVE_A TOR method beginning in May 2007 (U.S. EPA 2005b, 2006). All core CSN sites simultaneously changed in May 2007, while the method change was phased in over time after that date at supplemental CSN sites. NPACT used the IMPROVE_A TOR method exclusively.

Sampling protocol. The NPACT, CSN, and IMPROVE networks operated on different sampling schedules and used different sampling hardware. Whereas NPACT collected 2-week average samples, CSN/IMPROVE sites collected 24-hr average samples that were obtained every third day at all IMPROVE sites and at most core CSN sites, and every sixth day at supplemental CSN sites. The use of different sampling devices with different pump flow rates and blank correction methods may also contribute to data inconsistencies among monitoring networks.

*Exploratory data analysis for data comparability*. To assess data comparability between networks, we performed exploratory analyses by generating graphical displays (maps, scatter plots, and time-series plots) and summary statistics.

Sparse coverage in urban space. We investigated the potential impact of the number, density, and locations of monitors within each area on spatiotemporal prediction model estimates by assessing city-specific spatial distributions of monitors and comparing estimated temporal patterns between networks. The temporal patterns were estimated by smoothing time-series data across monitoring sites.

Different filter analysis protocols. We compared the two filter analysis methods for EC between the CSN and IMPROVE networks as well as within the CSN network. We compared pairs of daily average EC measurements collected from January 2000 through July 2007 at four co-located CSN and IMPROVE sites using the NIOSH TOT and IMPROVE_A TOR filter analysis methods, respectively. In addition, there were 2 months of overlap from early May to early July in 2007 when both NIOSH TOT and IMPROVE_A TOR methods were used at the same core CSN sites. We compared pairs of daily average EC measurements during the overlapping time period using two methods at the six core CSN sites co-located with NPACT fixed sites.

Different sampling protocols. Given that NPACT collected 2-week average measurements and CSN and IMPROVE collected 24-hr samples every third or sixth day, it was not clear whether CSN and IMPROVE data could reliably estimate 2-week averages and temporal trends. The majority of CSN and IMPROVE data available for NPACT were measurements taken every sixth day at supplemental CSN sites. There were relatively few network sites with data collected every third day within 200 km of a MESA city center, because there were only 54 core CSN sites in the United States, and IMPROVE sites are mostly distant from cities. Thus we investigated the importance of sampling frequency by making within-site comparisons at four of the six CSN sites co-located with NPACT fixed sites that collected data every third day. Specifically, we compared the smoothed temporal patterns of 2-week average silicon estimates using data obtained from every third-day samples versus a reduced subset of every sixth-day samples. In addition to different sampling frequencies, the impact of differences in sampling hardware systems was compared at all six co-located sites using pairs of 2-week averages for EC and silicon from CSN and NPACT. The comparison for EC was restricted to the period during and after May 2007 when the IMPROVE_A TOR filter analysis method was adopted at core CSN sites. All six CSN sites co-located with NPACT fixed sites were core sites.

*Exposure prediction model*. The NPACT exposure prediction model aimed to predict 2-week average concentrations of PM_2.5_ components at participant addresses by adopting the spatiotemporal modeling framework developed for the MESA Air study. Overall, NPACT monitoring sites provided reasonable spatial coverage of MESA cities (average of 3–10 sites/km for fixed and home-outdoor sites combined in each city). However, there were only three to seven fixed NPACT sites providing continuously collected data for each city (over 4 years for silicon or 18 months for EC), in contrast with the larger numbers of home-outdoor sites (87–116 per city) operating for only one to three 2-week periods. See Supplemental Material, Figure S1, for an illustration of the spatial and temporal resolution of the NPACT monitoring design in the Los Angeles area as an example. The spatiotemporal model was designed to effectively utilize such highly imbalanced monitoring data. Applications of the city-specific spatio-temporal models for PM_2.5_, nitrogen dioxide (NO_2_), nitrogen oxides (NO_x_), and black carbon in MESA Air have been described previously (Keller et al. 2015; Lindström et al. 2013b; Sampson et al. 2011; Szpiro et al. 2011) in situations where regulatory monitoring data were used to supplement the MESA Air campaign. The long time series of the regulatory monitoring data contributed to characterization of temporal features, whereas the MESA Air monitoring data enhanced the model at a relatively fine spatial scale. The model is available for implementation in the R package “SpatioTemporal” (Lindström et al. 2013a, 2013b). In brief, this model assumes that 2-week average space-time concentrations consist of site-specific long-term means, site-specific temporal trends, and spatiotemporal residuals. Long-term means and temporal trends vary over space as characterized by geographical predictors and spatial correlation structures. Temporal trends include spatially homogenous temporal trend functions scaled by spatially varying trend coefficients. Temporal trend functions are derived from a singular value decomposition of the data at sites with long time series before model fitting. Spatiotemporal residuals are assumed to be temporally independent but spatially dependent.

*Exploration of possible spatiotemporal modeling approaches*. We explored the possibility of fitting three approaches to develop city-specific spatiotemporal prediction models for silicon and EC based on our experience developing the MESA Air spatiotemporal model for PM_2.5_ (Keller et al. 2015). For this exploration, we used results of descriptive analyses described in the previous section (“Exploratory data analysis for data comparability”) and performed additional data analyses. First, we considered the full spatiotemporal model directly using all available PM_2.5_ component data from the regulatory and NPACT monitoring networks as in Keller et al. (2015) (Approach 1). In the PM_2.5_ spatiotemporal modeling work, the regulatory and MESA Air data were highly correlated and thus combined, allowing this rich data set to be used for the full model. The spatial density of PM_2.5_ component regulatory monitoring sites and the data comparability between networks are the criteria we considered to indicate the feasibility of Approach 1. In the event that the multiple sources of PM_2.5_ component data were insufficiently compatible to combine, NPACT data alone were too limited to support the full spatiotemporal model. To deal with such a case, we considered Approach 2 as a simplified version of the spatiotemporal model based only on NPACT data that assumed one temporal trend and without any spatial dependence structure. One homogeneous temporal trend in each city is a strong assumption. We investigated whether this assumption was appropriate by comparing a single temporal pattern estimated using fixed-site data for 4 years or 18 months with time-series data across about 50 home-outdoor sites in each city. Finally, we considered using the temporal trend functions estimated from other pollutant time series, such as PM_2.5_ and NO_x_, instead of those from PM_2.5_ components in the full spatiotemporal model framework (Approach 3). These pollutants have longer time series of data at many more regulatory monitoring sites than those of PM_2.5_ components in NPACT. Fitting the full spatiotemporal models using substituted trend functions in Approach 3 would be justified when there is good agreement between the two trend functions (i.e., the PM_2.5_/NO_x_ and the PM_2.5_ component trend functions). We compared the two temporal patterns between EC/silicon in NPACT and PM_2.5_/NO_x_ in the U.S. EPA AQS to assess the feasibility of Approach 3. Daily PM_2.5_ and NO_x_ data measured at the U.S. EPA monitoring sites located within 200 km of the six MESA cities were obtained from the AQS database and converted to 2-week averages.

## Results

Table 1 summarizes important characteristics of the PM_2.5_ component monitoring data across the NPACT, CSN, and IMPROVE networks. The table highlights three aspects of the regulatory and NPACT monitoring data that may make it difficult to combine the multiple sources in one unified spatio-temporal model: sparse spatial coverage, analysis method differences for carbon data, and different sampling protocols.

**Table 1:** Major contrasting characteristics among NPACT, CSN, and IMPROVE networks.

Characteristic	NPACT	CSN	IMPROVE
Sampling design
Location of sites	Urban	Urban	Rural
Spatial density in MESA city areas	Dense (92–112 sites in each city)	Sparse (8–27)	Sparse (1–8)
Monitoring period	2005–2009	Since 1999	Since 1987
Sampling schedule	2-week average	24-hr average: 1 in 3 or 6 day	24-hr average: 1 in 3 day
Filter analysis method
Analysis method for elements	XRF^*a*^	XRF	XRF
Analysis method for carbon	IMPROVE_A TOR^*a*^	NIOSH TOT IMPROVE_A TOR^*b*^	IMPROVE_A TOR
Blank correction using backup quartz filter	Yes	No	Yes
Sampling protocol
Sampler typefor elements	HPEM	Met One SASS,^*c*^ Andersen RAAS, URG MASS, and R&P	IMPROVE
Sampler typefor carbon	HPEM	Met One SASS,^*c*^ Andersen RAAS, URG MASS, R&P, and URG 3000N^*b*^	IMPROVE
Pump flow rate	1.8 L/min	6.7 ~ 16.7 L/min 22.8 L/min^*b*^	22.7 L/min
Abbreviations: Andersen RAAS, Andersen Reference Ambient Air Sampler; HPEM, Harvard Personal Environmental Monitor; Met One SASS, Met One Speciation Air Sampler System; R&P, Rupprecht and Patahnick; URG, University Research Glassworks. ^***a***^XRF analysis was performed at Cooper Environmental Services of Portland, Oregon, and IMPROVE_A TOR analysis was performed at Sunset Laboratory Inc. of Tigard, Oregon. ^***b***^New carbon sampling and analysis protocols have been implemented at core CSN sites since May 2007. ^***c***^Used in about 75% of CSN sites in 2006.			

**Figure 1:**
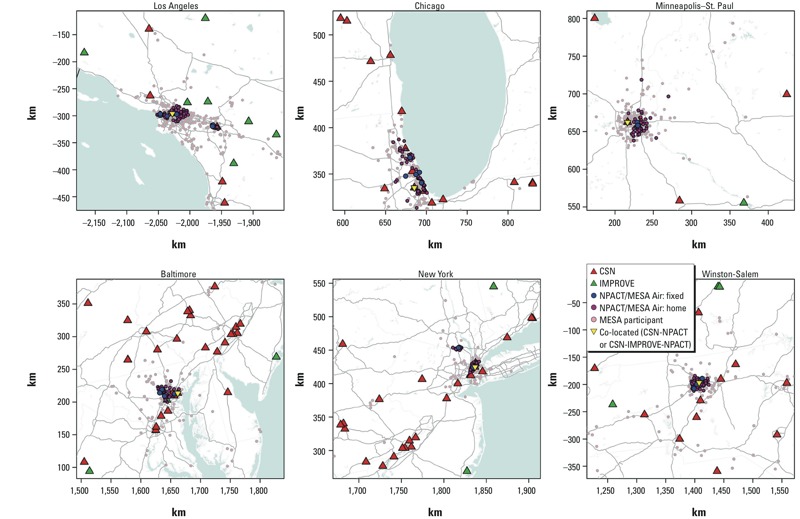
Locations of CSN, IMPROVE, and NPACT monitoring sites for PM_2.5_ components within 200 km from city centers in six MESA city areas. Each map is restricted to a smaller area including all monitoring sites than the 200-km buffer area from the city center; one to three IMPROVE sites in four cities are not shown because they are hidden behind many other sites in the city center areas or with co-located CSN sites.

*Data compatibility between CSN, IMPROVE, and NPACT networks*. Sparse coverage in urban space. There were 6–27 CSN and 1–8 IMPROVE monitoring sites within 200 km of each city center (Figure 1 and Table 2). However, MESA participant homes were clustered near the center of each area, whereas only a few CSN sites were close to the city center and most IMPROVE sites were located in rural areas away from participants. See Supplemental Material, Figure S2, for estimated smoothed temporal patterns for the CSN and IMPROVE sites in six city areas. The temporal patterns for EC at eight IMPROVE sites were different from those observed at six CSN sites in Los Angeles. There were also differences between the temporal patterns for silicon across networks, but these were less striking. In the other five city regions, the temporal patterns for EC were more or less heterogeneous depending on city, whereas those for silicon were relatively consistent in all cities.

**Table 2:** Number of sites with long-term monitoring data available within 200 km of six MESA city areas between 1999 and 2009.

Area	Total^*a*^	Regulatory CSN total	Regulatory CSN 3-day	Regulatory CSN 6-day	Regulatory IMPROVE^*b*^ total (3-day)	NPACT fixed total (14-day avg)	NPACT home-outdoor total (14-day avg)
Los Angeles	21 (137)^*c*^	6	3	3	8	7	116
Chicago	23 (122)	15	4	11	1	7	99
Minneapolis–St. Paul	10 (114)	6	2	4	1	3	104
Baltimore^*d*^	37 (124)	27	8	19	5	5	87
New York^*d*^	31 (138)	25	14	11	3	3	107
Winston-Salem	19 (111)	12	2	10	3	4	92
^***a***^Co-located sites are counted as multiple sites (two for CSN and NPACT or CSN and IMPROVE, and three for CSN, IMPROVE, and NPACT). ^***b***^The numbers of IMPROVE sites shown in Figure 1 are 7, 0, 1, 2, 2, and 3. One to three IMPROVE sites in four cities are not shown in Figure 2 because they are hidden behind many other sites in the city center areas or at sites co-located with CSN sites. ^***c***^Number of sites excluding NPACT–MESA Air home sites (number of sites including home sites). ^***d***^Thirteen sites appear in both Baltimore and New York due to overlap of regions: 12 CSN (3 for every-3rd-day and 9 for every-6th-day sampling sites, respectively) and 1 IMPROVE.							

**Figure 2:**
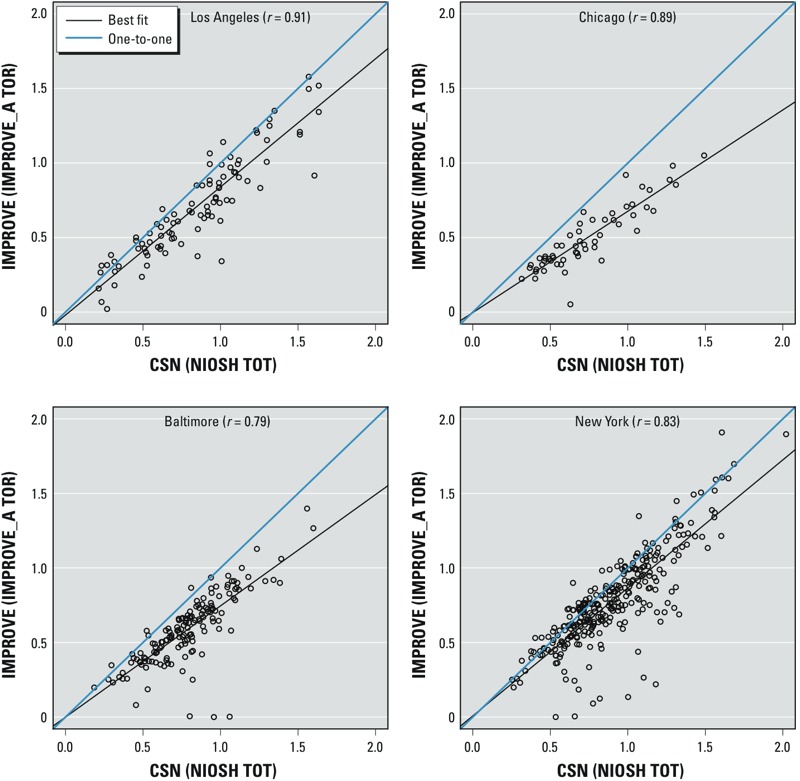
Scatter plots of log-transformed every-3rd-day measurements of EC (μg/m^3^) between CSN and IMPROVE at four co-located sites in Los Angeles, Chicago, Baltimore, and New York from January 2000 through July 2007.

Different filter analysis protocols. Although Figure 2 shows that at four co-located sites there was moderate to high agreement between protocols (correlation coefficients = 0.79–0.91), these are not consistently and sufficiently high to conclude that the data are exchangeable in some city areas for daily average measurements of EC collected from the CSN versus IMPROVE networks before the method change in May 2007. See Supplemental Material, Figure S3, for a comparison of 24-hr average measurements of EC between the NIOSH TOT and IMPROVE_A TOR filter analysis methods for the 2-month period of overlap at one CSN site in each MESA city region. In Chicago and New York, the two methods had obvious systematic differences indicated by best-fit lines with negative intercepts, even though they were highly correlated; correlation coefficients were 0.94 and 0.97, attributable partly to the large variability between measurements in these cities. In contrast, the other cities displayed weaker systematic differences and had moderate correlations (0.71–0.84).

**Figure 3:**
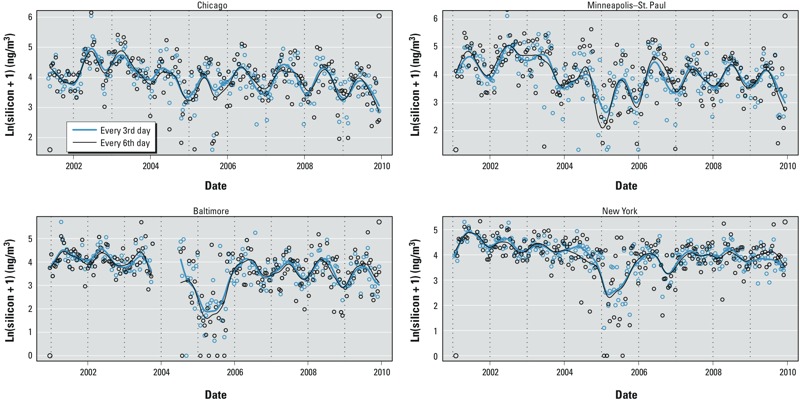
Time-series plots of log-transformed (Ln) 2-week averages of silicon between every-3rd-day and every-6th-day measurements at the same four CSN sites co-located with four NPACT fixed sites in Chicago, Minneapolis–St. Paul, Baltimore, and New York from 1999 to 2009.

**Figure 4:**
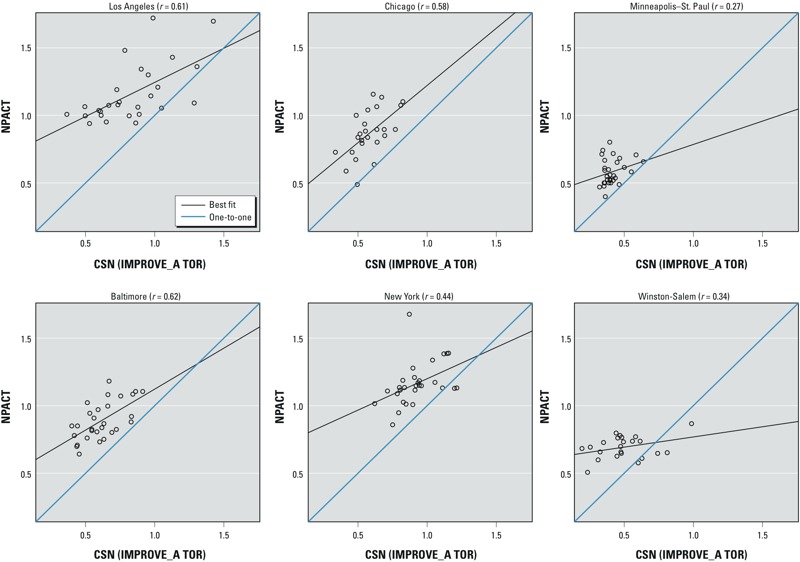
Scatter plots of log-transformed 2-week averages of EC (μg/m^3^) for the overlapping period from May 2007 through August 2008 between co-located CSN and NPACT fixed sites in each of six MESA city areas.

Different sampling protocols. Table 2 indicates numbers of CSN and IMPROVE sites by sampling schedule. Fewer than half of the CSN sites (the core CSN sites) and all the IMPROVE sites sampled PM_2.5_ components every third day, whereas more than half of the CSN sites (the supplemental sites) sampled every sixth day. Smoothed temporal patterns for 2-week averages of silicon based on CSN data collected at four sites co-located with NPACT fixed sites generally did not vary greatly when based on data collected every sixth day versus every third day at the same site, although a few local differences were evident (Figure 3). Correlations between 2-week average EC concentrations measured during May 2007–August 2008 at co-located NPACT fixed sites and CSN sites (using the IMPROVE_A TOR filter analysis method) in each city were relatively low (0.27–0.62) (Figure 4). In addition to NPACT measurements being generally higher than CSN measurements in all cities, there were nonsystematic differences indicated by some measurements being far from best-fit lines between the two networks. Time-series plots with smoothed temporal patterns of the same data used in Figure 4 show local differences over time (see also Supplemental Material, Figure S4). Supplemental Material, Figures S5 and S6, show that silicon measurements are more comparable than EC with higher correlation coefficients of 0.56–0.78.

*Possible exposure modeling approaches*. Approach 1: Full spatiotemporal models combining the CSN/IMPROVE and NPACT data. The regulatory monitoring data for PM_2.5_ components in each city region within a 200-km boundary (7–32 sites) were more limited than those for other pollutants such as PM_2.5_ in the much smaller area within 75 km of the city center (16–45 sites) (Table 2; see also Supplemental Material, Table S1). The descriptive analyses in the previous section (“Data compatibility between CSN, IMPROVE, and NPACT networks”) showed evidence of differences related to filter analysis methods and sampling protocols (Figures 2 and 4; see also Supplemental Material, Figures S4–S6). Thus, we concluded that NPACT data should not be combined with CSN and IMPROVE data to generate full spatiotemporal models for PM_2.5_ components for each city.

Approach 2: Simplified spatiotemporal models based on the NPACT data only. Based on a graphical analysis comparing the single temporal pattern from NPACT fixed site data with measurements from the home-outdoor sites in each city (as illustrated for Los Angeles and Chicago in Supplemental Material, Figure S7), we concluded that the single smoothed temporal patterns generally represented the temporal variability across home sites.

Approach 3: Full spatiotemporal models using another pollutant. From the comparison of estimated temporal patterns for PM_2.5_ and NO_x_ based on U.S. EPA site data with those for EC and silicon based on fixed site NPACT data, we concluded that the patterns did not tend to be consistent enough to support using other pollutant data to generate full spatiotemporal models for PM_2.5_ components (i.e., Approach 3). For example, temporal patterns for EC and silicon differed from those for PM_2.5_ and NO_x_ particularly in the Minneapolis and St. Paul area (see Supplemental Material, Figure S8).

## Discussion

We explored the features of regulatory and NPACT monitoring data for EC and silicon relevant to our goal of combining all available exposure data in spatiotemporal prediction models to investigate health effects of long-term exposures to PM_2.5_ chemical components in the NPACT study. The small number of CSN and IMPROVE regulatory monitoring sites deployed in NPACT study areas limited the amount of additional data available for modeling. In addition, we found insufficient between-network consistency to combine CSN, IMPROVE, and NPACT data in one spatiotemporal model. These findings led us to conclude that we should develop spatiotemporal models using NPACT monitoring data only. Given the limited space–time data in NPACT, the resulting spatiotemporal models needed to be simplified by assuming only a single temporal time trend in each study area.

We found inconsistencies between measurements from the NPACT and regulatory monitoring networks for both EC and silicon, even when both networks used the same filter analysis methods. Exploration of possible factors resulting in the inconsistency will help future studies that perform study-specific monitoring campaigns for PM_2.5_ components to supplement regulatory data for exposure prediction and subsequent health analysis. For EC, we believe that the inconsistency is attributable primarily to differences in sampling periods of 2-week versus daily samples in NPACT and CSN/IMPROVE, respectively (see Supplemental Material, “Sampling periods and EC measurements,” for detailed information). In addition to the sampling period, other differences in carbon sampling between the networks could have contributed to inconsistencies in the data. NPACT used a blank correction protocol based on backup quartz filters, whereas CSN did not apply blank corrections. Filter handling, transport, and storage in NPACT may also have introduced artifacts and resulted in differences in measurements between the two networks, despite our extensive quality assurance and control procedures. However, the good agreement between total carbon measurements in the CSN and NPACT networks (Vedal et al. 2013) suggests that the inconsistency of EC and OC measures between the two networks is more likely driven by the EC–OC split rather than the sampling and blank correction protocols.

Differences between silicon measurements from co-located NPACT and CSN monitors placed a few meters away from each other might be driven by microscale local plume gradients. Another possible explanation could be the use of different sampling equipment. Contamination of the filters by the silicon grease used in the HPEM sampler can result in increased silicon concentrations. However, grease contamination usually appears as very large spikes in contaminated samples compared with other samples; such spikes were not observed in our data (data not shown). Consistency between PM_2.5_ and sulfur concentrations measured by the co-located monitors (data not shown) suggest that the Teflon filters used by the two networks generally sampled the same fine particles.

Some studies have developed calibration models to allow combined analysis of data collected by CSN and IMPROVE networks. White (2008) and Malm et al. (2011) used elemental, organic, and total carbon data in 2005 and 2006 at 7–12 co-located urban CSN and IMPROVE sites over the continental United States to estimate relationships of EC between the two networks. Their IMPROVE-adjusted EC at CSN sites was highly correlated with EC at co-located IMPROVE sites (*R*^2^ = 0.80–0.94). However, these calibrations were based on data collected at a relatively small number of co-located sites during a short time period. More research is needed to determine whether these calibrations can be applied to other areas or years.

Unlike our study, other published studies of the health effects of long-term average PM_2.5_ component concentrations have relied exclusively on regulatory monitoring data. Ostro et al. (2010) used CSN data and assigned PM_2.5_ components at the nearest monitors to participant homes in California. Bergen et al. (2013) used CSN and IMPROVE data to build universal kriging models across the United States. Both studies used long-term averages and developed purely spatial models in large spatial domains. To take advantage of the extensive project-based monitoring campaigns designed to represent fine-scale spatial variability of PM_2.5_ component concentrations across the target cohort residences, the NPACT options were either to use the NPACT data alone or to combine the NPACT data with regulatory monitoring data.

Our findings suggest that it may be difficult to transfer existing spatiotemporal prediction modeling approaches developed for PM_2.5_ (Keller et al. 2015; Paciorek et al. 2009; Sampson et al. 2011; Yanosky et al. 2009) to modeling PM_2.5_ components. Several features of the PM_2.5_ component data make a direct transfer difficult. Although the regulatory PM_2.5_ monitoring data were collected under consistent protocols over a relatively long period since the 1990s and across about 1,000 monitoring locations in the United States (Hand et al. 2011; U.S. EPA 2004), this is not the case for PM_2.5_ component data. Furthermore, there is reasonable agreement for PM_2.5_, unlike for PM_2.5_ components, between these regulatory monitoring data and the data collected by community-based campaigns such as MESA Air (correlation coefficients = 0.77–0.96 at six co-located sites in six MESA city regions; data not shown). Thus, although Keller et al. (2015) and Sampson et al. (2011) were able to combine regulatory and MESA Air monitoring data in city-specific spatiotemporal predictive models of PM_2.5_, we were unable to take the same approach in NPACT. Instead, we used only the NPACT data in PM_2.5_ component prediction modeling in order to avoid introducing heterogeneity and bias into our results.

Given widespread scientific interest in understanding the associations between long-term air pollution exposure and health for multiple pollutants, it is important that we also acquire sufficient understanding of monitoring data features, which may in turn affect exposure predictions and the resulting health effect estimates. Methodological research has shown that features of the underlying exposure surface, exposure assessment design, and approaches to exposure modeling may all affect health effect estimates (Gryparis et al. 2009; Kim et al. 2009; Szpiro and Paciorek 2013; Szpiro et al. 2011). This study adds monitoring data from multiple sources as another feature that could affect exposure modeling for inference about health effects.

## Conclusions

U.S. regulatory monitoring data for PM_2.5_ components measured at CSN and IMPROVE sites are a potentially rich data resource to be used alone or combined with project-based monitoring data for the study of health effects of PM_2.5_ components. However, the sparse spatial coverage of these networks and differences across networks in the analysis and sampling protocols for some PM_2.5_ components could lead to biased or imprecise findings in health analyses, particularly if the data from different sources are combined without careful consideration. Future studies of long-term average concentrations of PM_2.5_ components and health need to assess exposure data characteristics before designing their own monitoring campaigns and developing exposure prediction models.

## Supplemental Material

(1.3 MB) PDF
