# Supplementary material for: Combining PM2.5 Component Data from Multiple Sources: Data Consistency and Characteristics Relevant to Epidemiological Analyses of Predicted Long-Term Exposures
Source: Environ Health Perspect. 2015 Feb 27;123(7):651–8. doi: 10.1289/ehp.1307744 (PMC4492258; doi:10.1289/ehp.1307744)
Supplement: (1.3 MB) PDF [file ehp.1307744.s001.acco.pdf]

Environ Health Perspect
doi: 10.1289/ehp.1307744

Note to Readers: *EHP* strives to ensure that all journal content is accessible to all readers. However, some figures and Supplemental Material published in *EHP* articles may not conform to 508 standards due to the complexity of the information being presented. If you need assistance accessing journal content, please contact ehp508@niehs.nih.gov. Our staff will work with you to assess and meet your accessibility needs within 3 working days.

Supplemental Material

Combining $PM_{2.5}$ Component Data from Multiple Sources: Data Consistency and Characteristics Relevant to Epidemiological Analyses of Predicted Long-Term Exposures

Sun-Young Kim, Lianne Sheppard, Timothy V. Larson, Joel D. Kaufman, and Sverre Vedal

Table of Contents

Sampling periods and EC measurements

Table S1. Numbers of monitoring sites and 2-week average samples for silicon, EC, and $PM_{2.5}$ used for spatio-temporal exposure prediction models by monitor type and city region in EPA AQS and NPACT monitoring networks from 2000 through 2009.

Figure S1. Temporal and spatial sampling for silicon and EC by CSN, IMPROVE, and NPACT monitors in Los Angeles. CSN sites changed the filter analysis method for EC an OC from the NIOSH_TOT (red dots) to IMPROVE_A TOR (orange dots) method from May 2007.

Figure S2. Temporal trends of log-transformed 2-week averages of silicon and EC measured by 6-27 CSN and 1-8 IMPROVE sites in Los Angeles, Chicago, Minneapolis-St. Paul, Baltimore, New York, and Winston-Salem from 1999 through 2009.

Figure S3. Scatter plots of log-transformed every 3rd day measurements of EC ($\mu g/m^3$) between pre- and post- filter analysis method change for the overlapping 2 months from May 2007 through July 2007 at six CSN sites co-located with NPACT sites in six MESA city areas.

Figure S4. Temporal trends of log-transformed 2-week average of EC for the overlapping period from May 2007 through August 2008 between co-located CSN and NPACT fixed sites in each of six MESA city areas.

Figure S5. Scatter plots of log-transformed 2-week averages of silicon for the overlapping period from August 2005 through August 2009 between co-located CSN and NPACT fixed sites in each of six MESA city areas.

Figure S6. Temporal trends of log-transformed 2-week averages of silicon for the overlapping period from August 2005 through August 2009 between co-located CSN and NPACT fixed sites in each of six MESA city areas.

Figure S7. Time series of log-transformed 2-week averages of silicon and EC across home-outdoor sites along with one temporal pattern estimated using NPACT fixed sites in Los Angeles and Chicago.

Figure S8. Temporal patterns of log-transformed 2-week averages of silicon (top) and EC (bottom) across NPACT fixed sites along with trends of $PM_{2.5}$ and NOx across EPA AQS sites in the Minneapolis-St. Paul area.

References

Sampling periods and EC measurements

Using Harvard Personal Environmental Monitor (HPEM) samplers with a low pump flow rate, NPACT sampled $PM_{2.5}$ components including EC for a sampling period of 2 weeks to collect sufficient amount of pollutants, while the sampling period was 24 hours given high volume samplers in CSN/IMPROVE. In the present analysis, EC concentrations measured at NPACT sites tended to be higher than EC concentrations measured at CSN sites; in contrast, Vedal et al. (2013) reported that OC concentrations measured at NPACT sites were lower than corresponding CSN measurements. It is possible that the more reactive OC components oxidized over two weeks (Kessler et al. 2012), thus resulting in decreased OC concentrations in NPACT when measured in the lab analysis. On the other hand, unpyrolized organics possibly formed by OC oxidation could have been quantified as EC fractions instead of OC fractions in the lab analysis, thus contributing to relatively higher EC concentrations (Cheng et al. 2010; Subramanian et al. 2006). In addition to the sampling period, other differences in carbon sampling between the NPACT and CSN/IMPROVE networks could have contributed to inconsistencies in the data. NPACT used the HPEM sampler with a lower pump flow rate and a blank correction protocol based on backup quartz filters. Filter handling, transport, and storage in NPACT may also have introduced differences in measurements between the two networks, even despite our extensive quality assurance and control procedures. However, the good agreement between total carbon measurements in the CSN and NPACT networks (Vedal et al. 2013) suggests that the inconsistency of EC and OC measures between the two networks is more likely driven by the EC-OC split rather than the sampling and blank correction protocols.

Table S1. Numbers of monitoring sites and 2-week average samples for silicon, EC, and $PM_{2.5}$ used for spatio-temporal exposure prediction models by monitor type and city region in EPA AQS and NPACT monitoring networks from 2000 through 2009.

Type	Silicon No. sites	Silicon Observation/site (min-max)	EC No. sites	EC Observation/site (min-max)	$PM_{2.5}$[a] No. sites	$PM_{2.5}$[a] Observation/site (min-max)
**Los Angeles**						
EPA AQS[b]	NA	NA	NA	NA	24	82-342
NACT/MESA Air fixed[c]	7	73-81	7	74-84	7	76-85
NACT/MESA Air home[c]	113	1-2	116	1-2	120	1-2
**Chicago**						
EPA AQS	NA	NA	NA	NA	20	71-320
NACT/MESA Air fixed	7	6-87	7	6-89	7	6-87
NACT/MESA Air home	99	1-3	99	1-3	136	1-4
**Minneapolis-St. Paul**						
EPA AQS	NA	NA	NA	NA	13	55-305
NACT/MESA Air fixed	3	79-86	3	79-86	3	81-89
NACT/MESA Air home	104	1-3	104	1-3	126	1-5
**Baltimore**						
EPA AQS	NA	NA	NA	NA	29	64-345
NACT/MESA Air fixed	5	18-85	5	18-86	5	18-92
NACT/MESA Air home	86	1-3	87	1-3	86	1-3
**New York**						
EPA AQS	NA	NA	NA	NA	45	51-342
NACT/MESA Air fixed	3	49-83	3	53-87	3	49-83
NACT/MESA Air home	107	1-3	107	1-3	107	1-3
**Winston-Salem**						
EPA AQS	NA	NA	NA	NA	16	86-346
NACT/MESA Air fixed	4	79-92	4	82-94	4	80-93
NACT/MESA Air home	92	1-3	92	1-3	114	1-4

Abbreviations: max, maximum; min, minimum; NA, Not available; AQS: Air Quality System.

[a]Numbers of EPA AQS and NPACT/MESA Air monitoring sites for $PM_{2.5}$ were obtained from Keller et al. 2014. [b]The six MESA city regions for selecting EPA AQS monitoring sites were defined by 200 km and 75 km buffers from the city centers for silicon/EC and $PM_{2.5}$ (Keller et al. 2014), respectively. [c]Silicon and $PM_{2.5}$ sampling for 2005-2009 and EC sampling for 2008-2009, respectively.

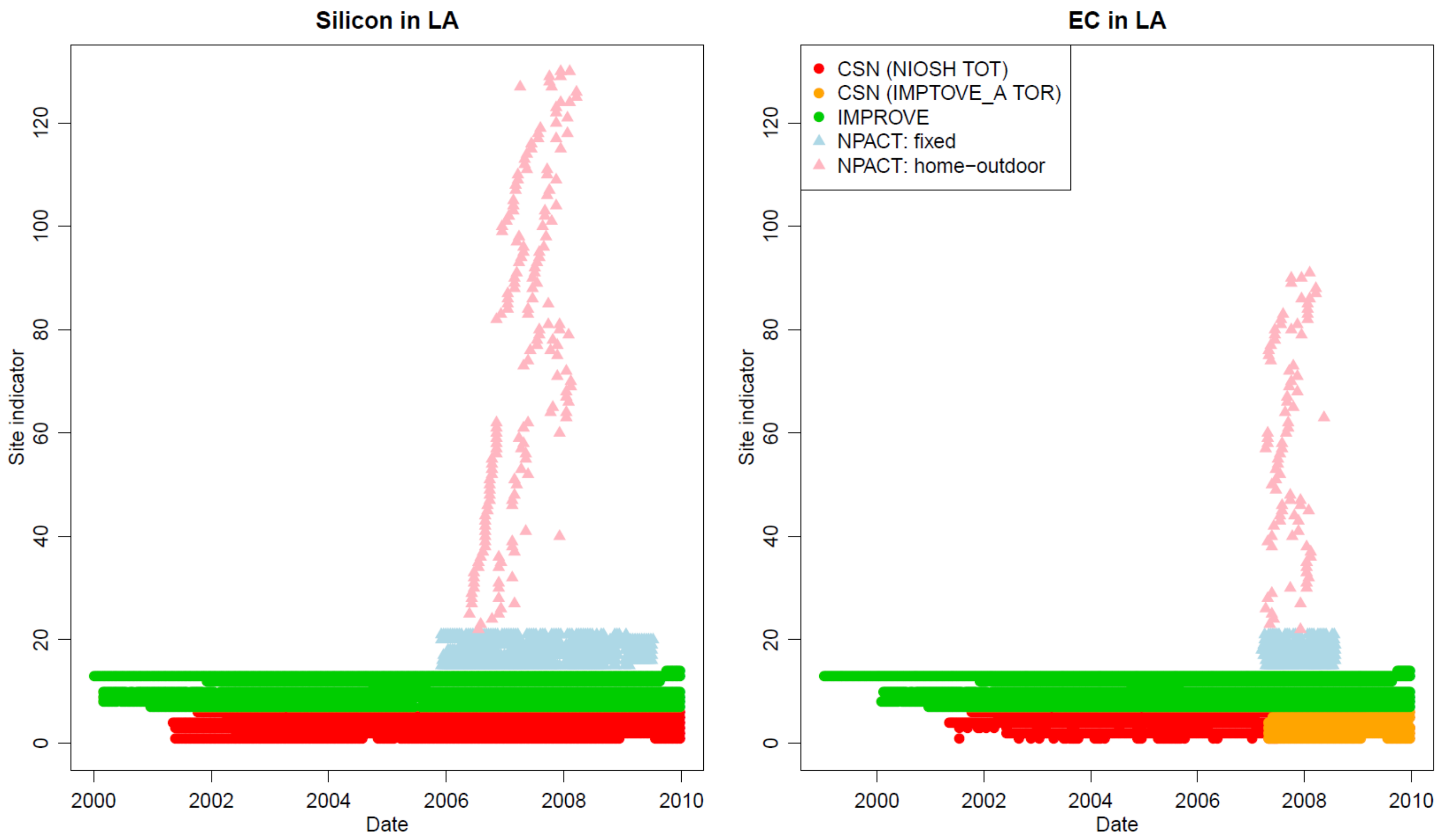

Figure S1. Temporal and spatial sampling for silicon and EC by CSN, IMPROVE, and NPACT monitors in Los Angeles. CSN sites changed the filter analysis method for EC an OC from the NIOSH_TOT (red dots) to IMPROVE_A TOR (orange dots) method from May 2007.

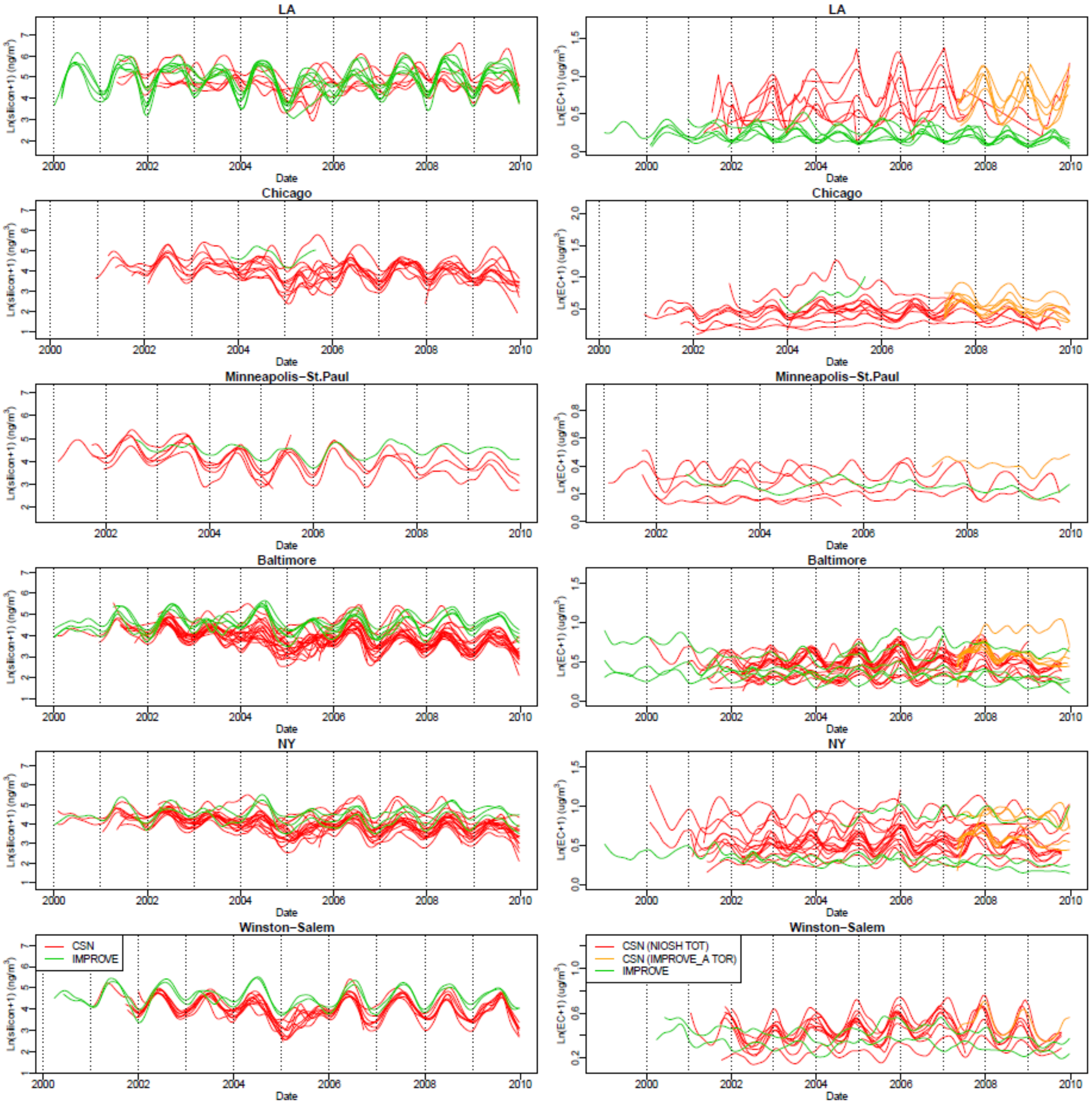

Figure S2. Temporal trends of log-transformed 2-week averages of silicon and EC measured by 6-27 CSN and 1-8 IMPROVE sites in Los Angeles, Chicago, Minneapolis-St. Paul, Baltimore, New York, and Winston-Salem from 1999 through 2009.

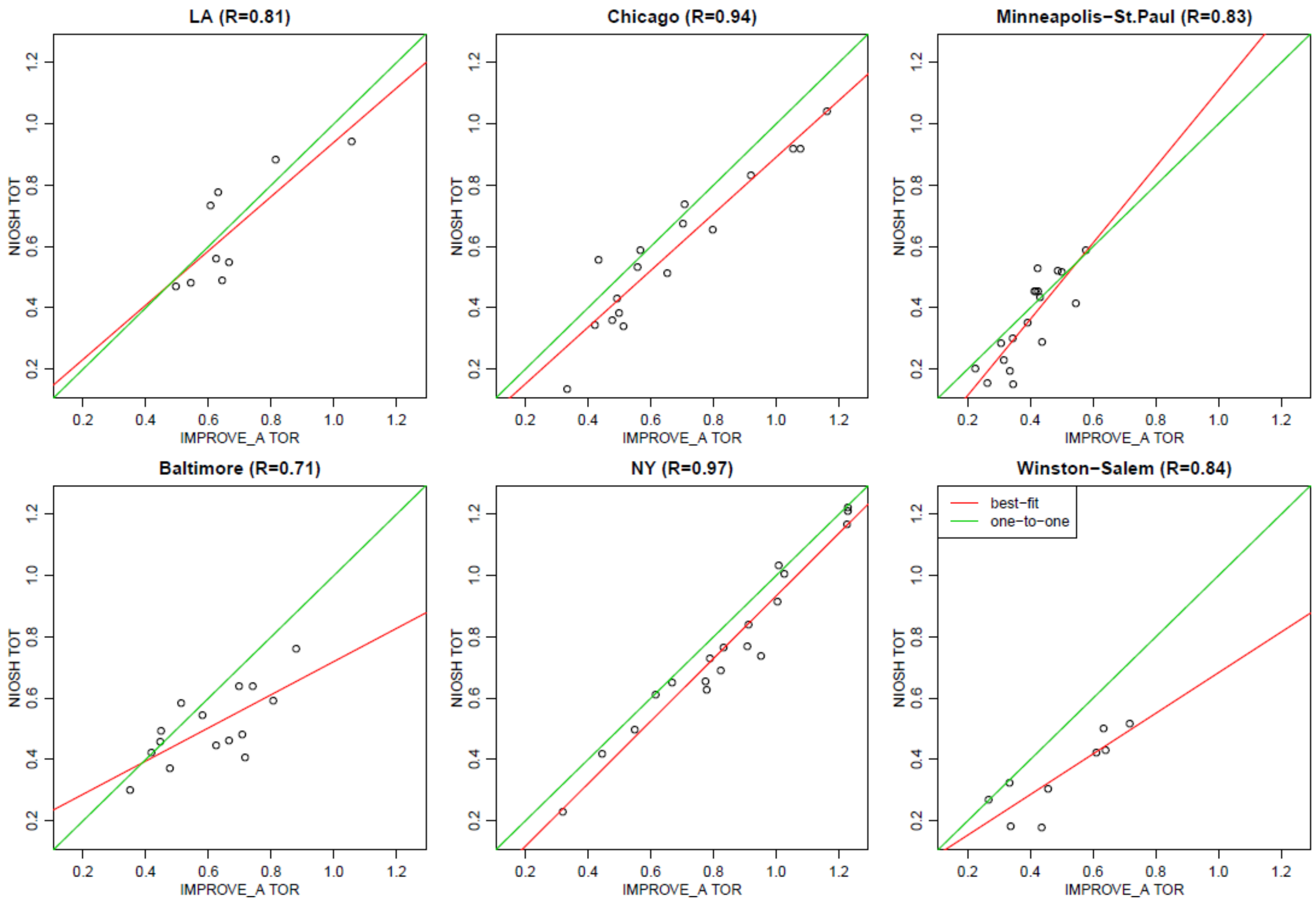

Figure S3. Scatter plots of log-transformed every 3rd day measurements of EC (μg/m^3) between pre- and post- filter analysis method change for the overlapping 2 months from May 2007 through July 2007 at six CSN sites co-located with NPACT sites in six MESA city areas.

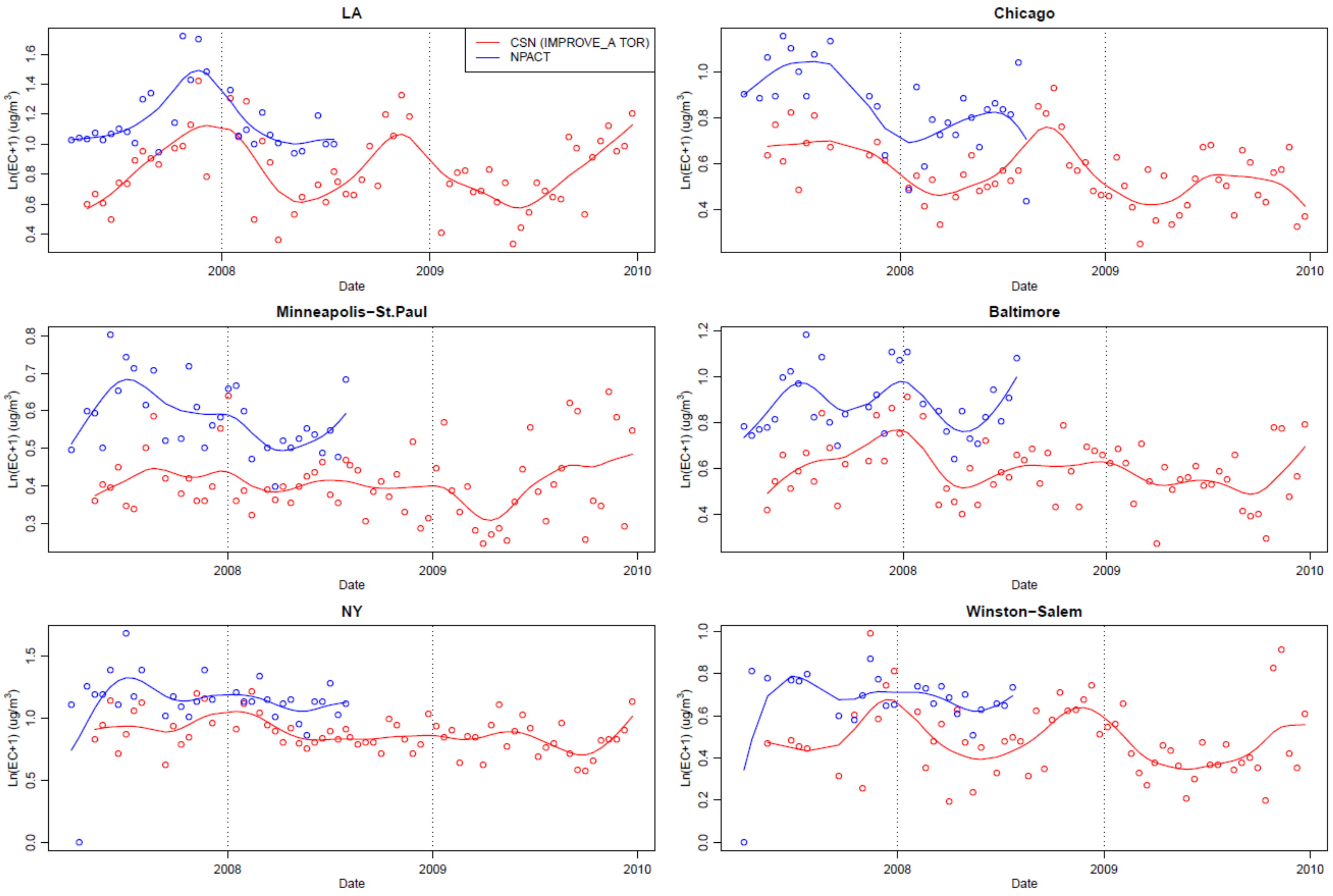

Figure S4. Temporal trends of log-transformed 2-week average of EC for the overlapping period from May 2007 through August 2008 between co-located CSN and NPACT fixed sites in each of six MESA city areas.

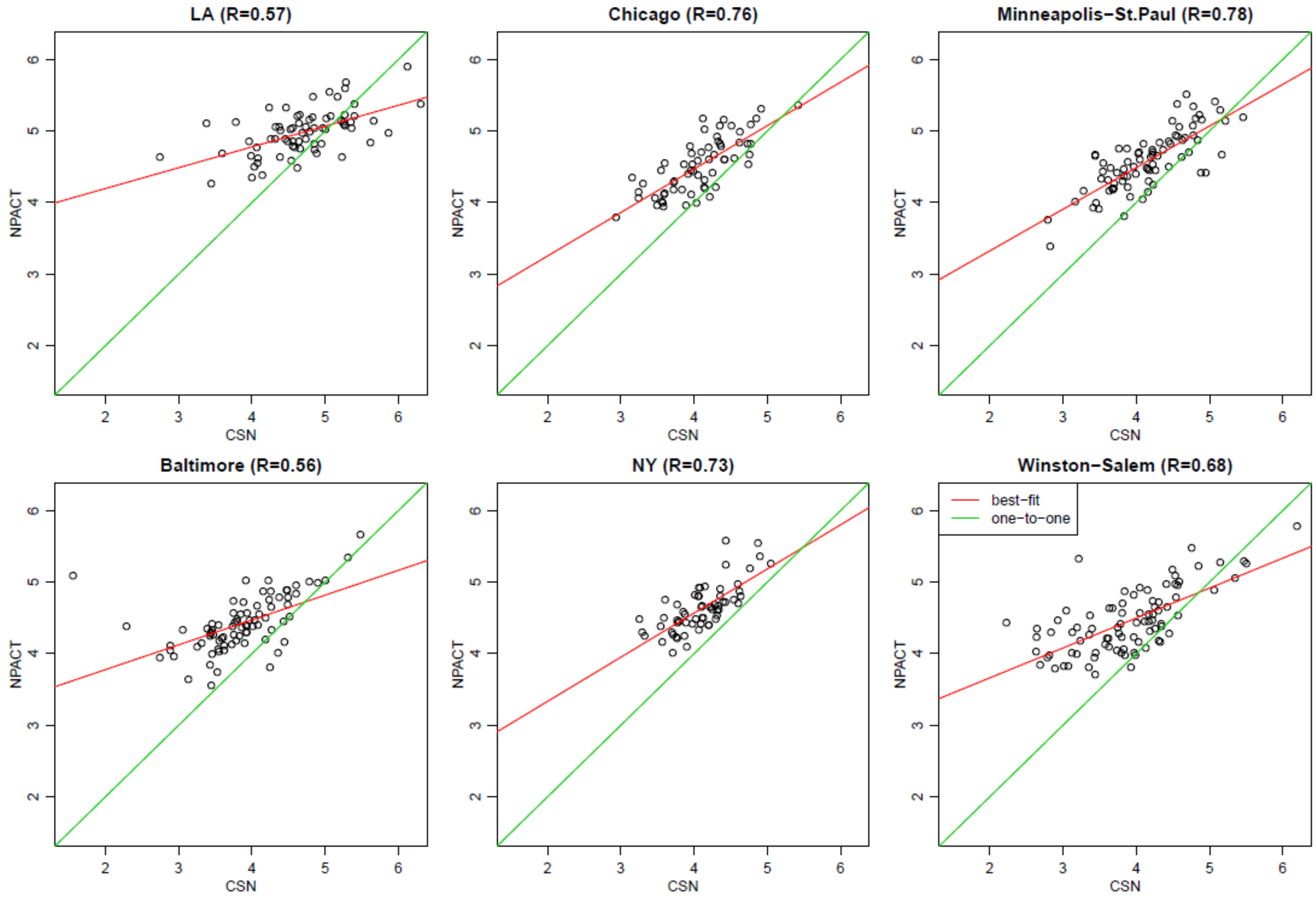

Figure S5. Scatter plots of log-transformed 2-week averages of silicon for the overlapping period from August 2005 through August 2009 between co-located CSN and NPACT fixed sites in each of six MESA city areas.

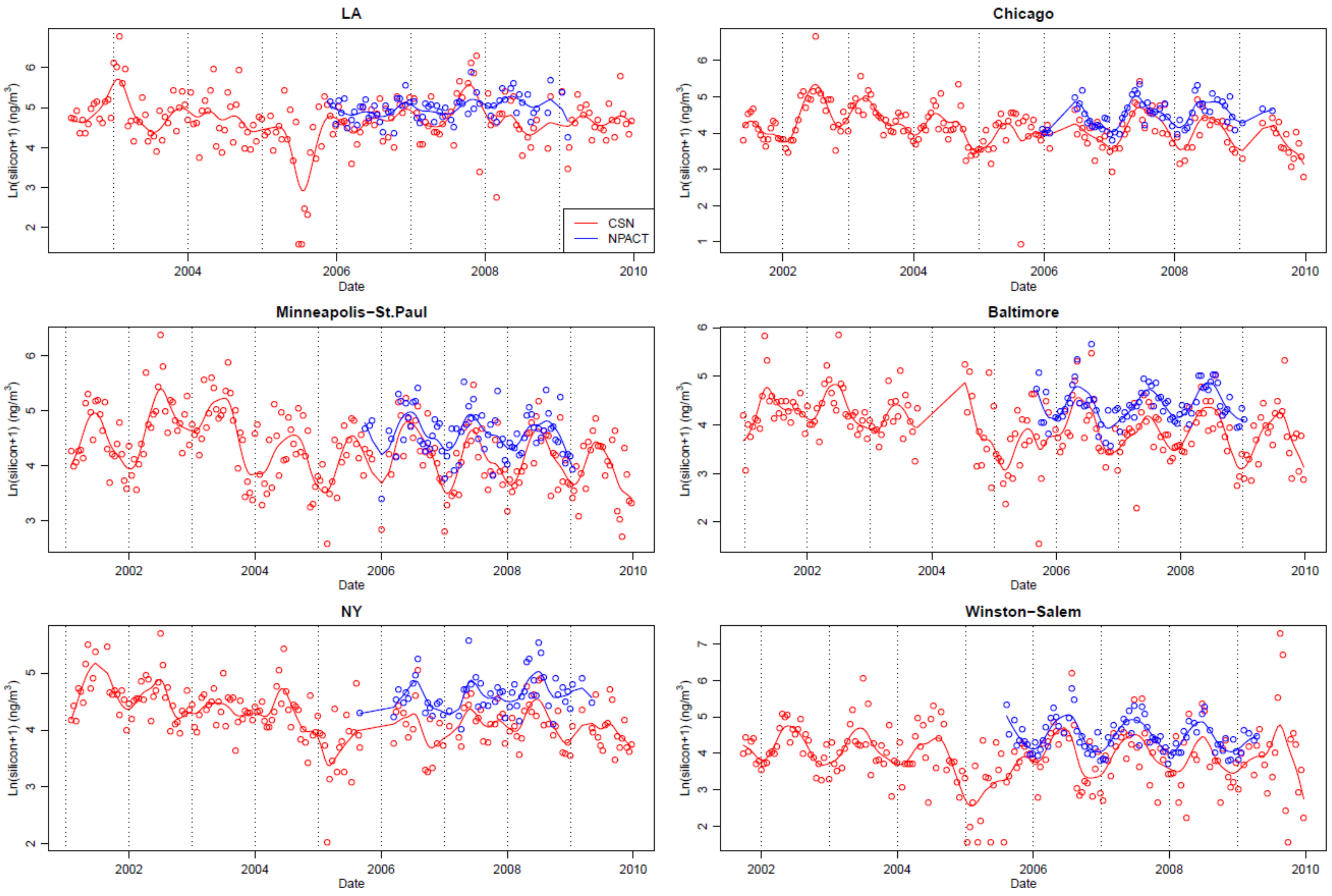

Figure S6. Temporal trends of log-transformed 2-week averages of silicon for the overlapping period from August 2005 through August 2009 between co-located CSN and NPACT fixed sites in each of six MESA city areas.

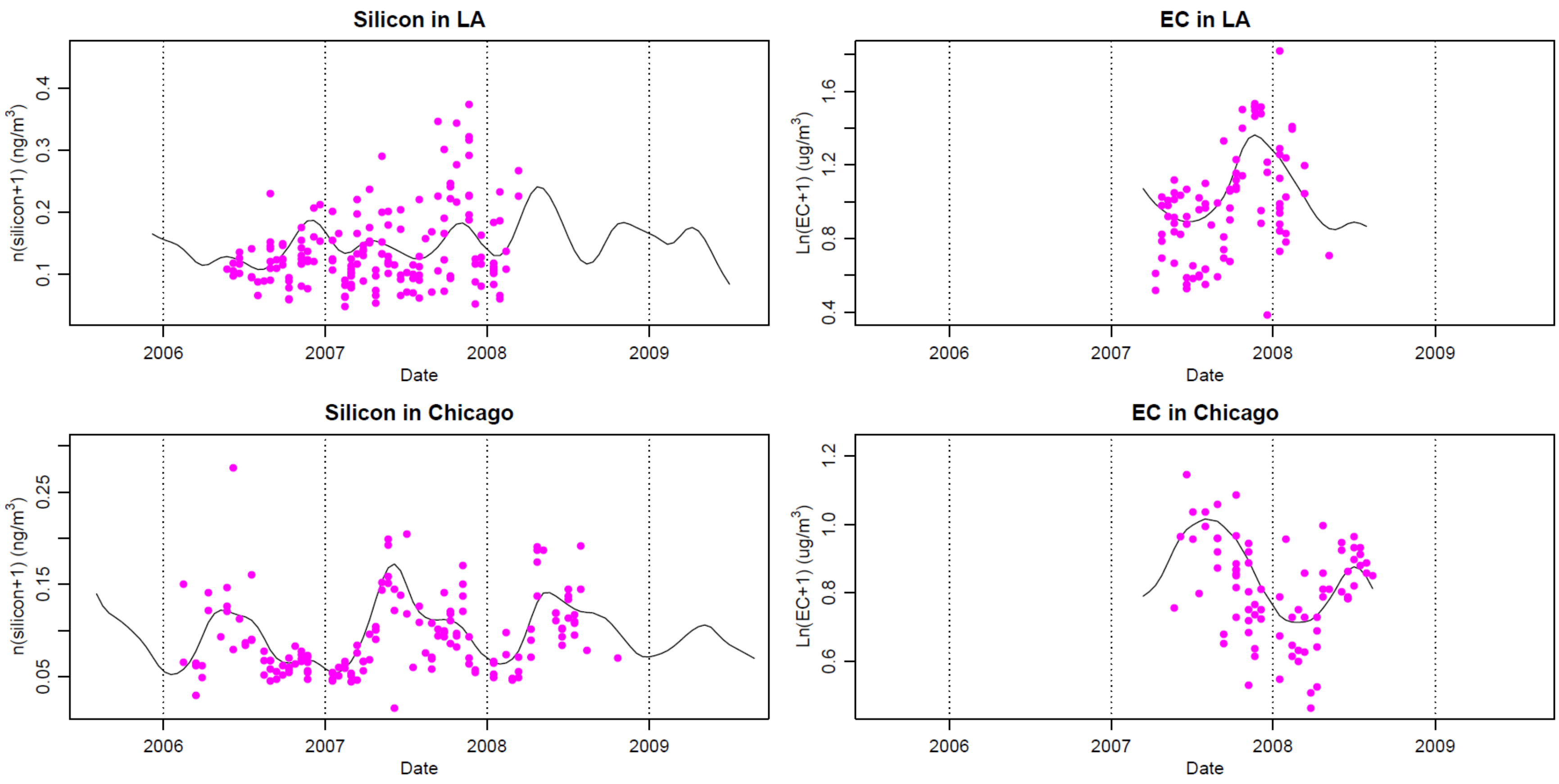

Figure S7. Time series of log-transformed 2-week averages of silicon and EC across home-outdoor sites along with one temporal pattern estimated using NPACT fixed sites in Los Angeles and Chicago.

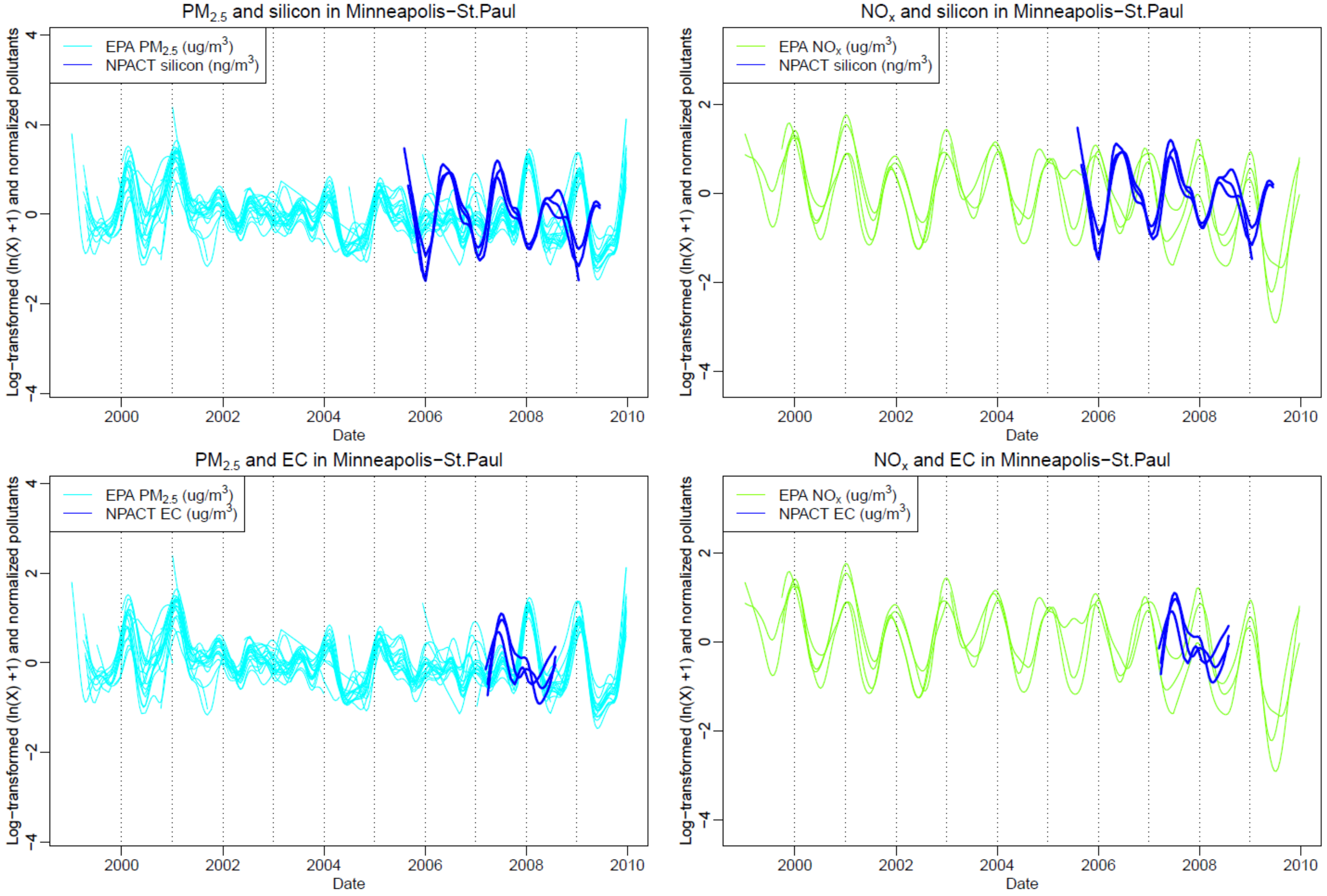

Figure S8. Temporal patterns of log-transformed 2-week averages of silicon (top) and EC (bottom) across NPACT fixed sites along with trends of $PM_{2.5}$ and NOx across EPA AQS sites in the Minneapolis-St. Paul area.

References

Cheng Y, He KB, Duan FK, Zheng M, Ma YL, Tan JH, et al. 2010. Improved measurement of carbonaceous aerosol: Evaluation of the sampling artifacts and intercomparison of the thermal-optical analysis methods. Atmos Chem Phys 10:8533–8548.

Keller JP, Olives C, Kim SY, Sheppard L, Sampson PD, Szpiro AA, et al. 2014. A unified spatiotemporal modeling approach for prediction of multiple air pollutants in the Multi-Ethnic Study of Atherosclerosis and Air Pollution. Environ Health Perspect (in press).

Kessler SH, Nah T, Daumit KE, Smith JD, Leone SR, Kolb CE, et al. 2012. OH-initiated heterogeneous aging of highly oxidized organic aerosol. J Phys Chem A 116:6358–6365.

Subramanian R, Khlystov AY, Robinson AL. 2006. Effect of peak inert-mode temperature on elemental carbon measured using thermal-optical analysis. Aerosol Sci Tech 40:763–780.

Vedal S, Kim SY, Miller KA, Fox JR, Bergen S, Gould T, et al. 2013. NPACT epidemiologic study of components of fine particulate matter and cardiovascular disease in the MESA and WHI-OS cohorts. Research Report 178. Health Effects Institute, Boston, MA
